# Getting DNA copy numbers without control samples

**DOI:** 10.1186/1748-7188-7-19

**Published:** 2012-08-16

**Authors:** Maria Ortiz-Estevez, Ander Aramburu, Angel Rubio

**Affiliations:** 1 Group of Bioinformatics, CEIT and TECNUN, University of Navarra, San Sebastian, Spain; 2 Computational Biology Group, Celgene Institute for Translational Research Europe (CITRE), Sevilla, Spain

**Keywords:** SNPs, Normalization, Scaling, High-throughput data, Batch effects

## Abstract

**Background:**

The selection of the reference to scale the data in a copy number analysis has paramount importance to achieve accurate estimates. Usually this reference is generated using control samples included in the study. However, these control samples are not always available and in these cases, an artificial reference must be created. A proper generation of this signal is crucial in terms of both noise and bias.

We propose NSA (Normality Search Algorithm), a scaling method that works with and without control samples. It is based on the assumption that genomic regions enriched in SNPs with identical copy numbers in both alleles are likely to be normal. These normal regions are predicted for each sample individually and used to calculate the final reference signal. NSA can be applied to any CN data regardless the microarray technology and preprocessing method. It also finds an optimal weighting of the samples minimizing possible batch effects.

**Results:**

Five human datasets (a subset of HapMap samples, Glioblastoma Multiforme (GBM), Ovarian, Prostate and Lung Cancer experiments) have been analyzed. It is shown that using only tumoral samples, NSA is able to remove the bias in the copy number estimation, to reduce the noise and therefore, to increase the ability to detect copy number aberrations (CNAs). These improvements allow NSA to also detect recurrent aberrations more accurately than other state of the art methods.

**Conclusions:**

NSA provides a robust and accurate reference for scaling probe signals data to CN values without the need of control samples. It minimizes the problems of bias, noise and batch effects in the estimation of CNs. Therefore, NSA scaling approach helps to better detect recurrent CNAs than current methods. The automatic selection of references makes it useful to perform bulk analysis of many GEO or ArrayExpress experiments without the need of developing a parser to find the normal samples or possible batches within the data. The method is available in the open-source R package NSA, which is an add-on to the aroma.cn framework. http://www.aroma-project.org/addons.

## Background

A DNA copy number aberration (CNA) is a pathological amplification or deletion of a part of the genome (a chromosome, one of their arms or a segment) which has been related to cancer development. In CNAs, DNA copy numbers (CNs) may be larger (gains and amplifications) or smaller (deletions and homozygous deletions) than the normal state (CN = 2).

CNAs can be measured using single nucleotide polymorphism (SNP) arrays. Although the initial application of these arrays was genotyping, they can also be used to calculate the CN estimates. Besides, regions with LOH (Loss of heterozygosity), which are zones of the genome that show no heterozygous SNPs, can be found using these arrays.

The number of SNPs in the arrays range from the initial ones which interrogate around 10,000 SNPs, to the newest ones which interrogate several millions of SNPs. The GWS arrays from Affymetrix, in addition to SNP probes, include non-polymorphic probes (known as CN probes) for analysis of Copy Number Variations (CNVs). In order to deal with Affymetrix SNP arrays it is required to apply several low level processes, namely, background removal, calibration, normalization and summarization [1]. The final results from these steps are two values (*θ*^*A*^ and *θ*^*B*^) for each SNP probeset which are approximately proportional to the number of copies of each allele. On the other hand, the CN probes of the latest arrays have a single value (*θ*^*T*^) proportional to the total number of copies. The proportionality constant is unknown and different for each SNP probeset and CN probe.

As previously stated, CNAs occur in segments of the genome. In order to find these aberrated regions, it is necessary to compute the scale factor which relates summarized SNP signals (*θ*^*A*^ and *θ*^*B*^) and CN values (*CN*^*A*^ and *CN*^*B*^) for each SNP. If there are control samples in the study, the computation of the scale factor is straighforward: two over a robust average of (*θ*^*A*^ + *θ*^*B*^) in the control samples [2-6] .

In this work it is assumed that a control sample has neutral copy number with no LOH in its whole genome. Of course, there can be CNVs in a control sample but, for sake of clarity, they are not considered here.

Unfortunately, there are many experiments that do not include control samples due to the difficulties to find them or simply to reduce experimental costs. In these cases, researchers opt for either using control samples from a public dataset or calculating a robust reference using the tumoral samples available in the experiment (implicitly assuming that for each SNP most of the tumoral samples have neutral CNs). However, as it will be shown, using samples from different labs can increase the noise in the CN estimations and assuming that SNPs have neutral CNs in most of the samples, although usually works, can introduce bias in the copy number estimations hiding real CNAs or even creating false ones, mostly when there is a recurrent aberration.

We propose an algorithm termed NSA (Normality Search Algorithm) that generates for each sample the corresponding reference without the need of control samples. Within each of the samples (control or tumoral) NSA detects regions with neutral CNs (CN = 2) and no LOH. Using these normal regions, the reference is calculated and used to scale the data through both SNPs and samples. We will show that NSA is able to correctly scale the CN estimates even if there are no control samples in the experiment. In addition, NSA is able to infer the batches within an experiment and find a proper weighting (different for each sample) to calculate the references.

The core of the NSA algorithm is the detection of normal regions in the genome. The developed method is based on the comparison of the signals for both alleles in each SNP. Heterozygous neutral copy number SNPs (HNCNs) have similar signals for both alleles ( $\theta_{j,i}^{A}≃\theta_{j,i}^{B}$) and their total copy number is two. For the majority of aberrations (amplifications, deletions or LOH), one of the alleles will have a larger signal than the other since the number of copies is different for each of them. AABB (or AAABBB) genotypes are very unlikely (except in multiploid cells) since it implies an amplification of both chromosomes and this occurs much less often than aberrations that involve only one of the chromosomes [7]. The nuclear assumption of NSA is that a SNP that have similar signals in both alleles ( $\theta_{j,i}^{A}≃\theta_{j,i}^{B}$) is probably a HNCN. NSA assumes that regions enriched in SNPs with similar signal for both alleles have neutral total CNs and no LOH.

Once NSA has inferred the normal regions, it also computes the optimal weights to calculate the reference using a weighted median. These weights are different for each sample and strongly related with the batches (set of samples that share some characteristics such day of hybridization, lab, person, etc) in which the samples were hybridized. Finally, the reference is calculated and the data scaled across both SNPs and samples.

NSA has been implemented in the aroma.cn framework and memory requirements are modest even for a large number of samples. Moreover, it is independent of both the preprocessing method or the microarray technology.

## Implementation

NSA is a population-based multi-array method for scaling any SNP & CN array technology, e.g. Affymetrix and Illumina. It identifies the normal regions within the samples, finds optimal weights to account for hybridization batches, calculates the corresponding references and, finally, performs a two-dimensional scaling.

### Data

We applied NSA to five different datasets. The first one is a subset of a Gliobastoma Multiforme (GBM) experiment that includes 64 tumoral samples [8] hybridized to Affymetrix Mapping 50K_Xba array. The second one is a Prostate Cancer analysis with 20 tumoral and 20 control samples [9] hybridized to Affymetrix Mapping 250K_Nsp array, the third one is a subset of 50 Lung Cancer and 20 control samples from [10] hybridized to Affymetrix GWS 6.0 array. The fourth one is a subset of an Ovarian Cancer experiment that includes 72 tumoral samples and 57 control samples [11] and, finally, the fifth one is a subset of HapMap samples [12]. It is shown here that, for these datasets, NSA provides more accurate and precise CN estimates than other state of the art scaling methods.

The input data of NSA are the summarized probe signals (*θ*^*A*^ and *θ*^*B*^) calculated using any summarization method such as dChip [13], RMA [14], CRMA v2 [1], ACNE [15], CalMaTe [16] for Affymetrix arrays or the one developed by [17] for Illumina technology. From these allele specific probe signals the fractions of the B-allele (*β *=* θ*^*B*^/(*θ*^*A*^ + *θ*^*B*^)) are obtained. CRMA v2 pre-processing methods have been applied to all the datasets. In the summarization step, ACNE (for Affymetrix Mapping 50 and 250K arrays) and CalMaTe (for the GWS6.0 arrays) were chosen because provide more accurate allele specific CNs.

### Detection of neutral DNA copy number regions

The main assumption of NSA is that gains of both chromosomes in a tumor sample are very unlikely to occur. It is shown in [7] that (for GBM) only 3% of the aberrations occur in both chromosomes. Therefore, we consider that if both *θ*^*A*^ and *θ*^*B*^ are approximately equal, the SNP is likely to be heterozygous with CN equal to 2 (i.e. it is a HNCN). Since CNAs occur in segments of the genome, the homozygous SNPs within a region enriched in HNCNs will probably also have neutral CNs. In order to quantify how similar the signals of both alleles are, we use the term *level of heterozygosity* (**LH**) which stands for a continuous approximation to the heterozygosity of a SNP (notice that this *level of heterozygosity* does not have anything to do with the same term in population genetics).

#### Level of Heterozygosity

By definition the **LH **for a given SNP *j* (j=1…J) in a sample *i* (i=1…I) is calculated as

(1) $${\text{LH}}_{j,i}=2\text{min}(\theta_{j,i}^{A},\theta_{j,i}^{B})/(\theta_{j,i}^{A}+\theta_{j,i}^{B})$$

where $\theta_{j,i}^{A}$ and $\theta_{j,i}^{B}$ are the corresponding signals of A and B alleles for SNP *j* in sample *i* and they are expected to be proportional to their CN values ( $CN_{j,i}^{A}$ and $CN_{j,i}^{B}$). If **LH**_*j*,*i*_ is close to 1 ( $\theta_{j,i}^{A}≃\theta_{j,i}^{B}$), SNP *j* is expected to be heterozygous in sample *i*. On the other hand, if it is close to 0 it means that one of the signals $\theta_{j,i}^{A}$ or $\theta_{j,i}^{B}$ is close to zero and, therefore, either the SNP *j* is homozygous in sample *i*, or that there are more copies of one allele than of the other (which occurs in an amplified zone). The values of the **LH** matrix (dimensions JxI) are within 0 and 1. Alternatively, **LH **can be defined as

(2) $${\text{LH}}_{j,i}=2\text{min}(\beta_{j,i},1-\beta_{j,i})$$

where $\beta_{j,i}=\theta_{j,i}^{B}/(\theta_{j,i}^{A}+\theta_{j,i}^{B})$ are the fractions of the B-allele.

**Figure 1:**
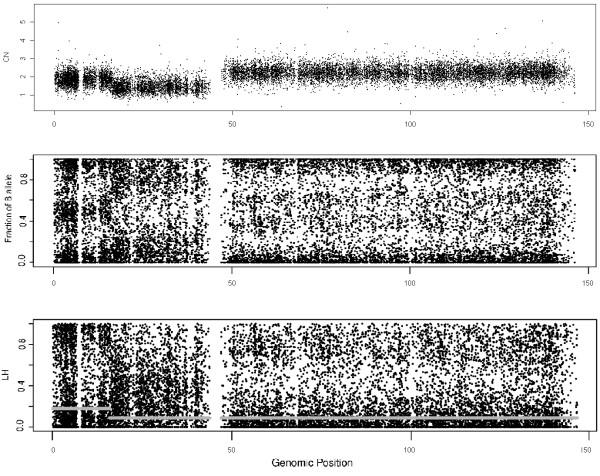
**These figures show the DNA copy number, the fraction of B allele and the LH values for chromosome 8 in sample GSM318736 from the Prostate Cancer dataset hybridized to Affymetrix Mapping250K_Nsp.** Heterozygous SNPs tend to have larger LH values (closer to 1) than homozygous SNPs (LH values closer to 0). The thick gray line over the LH figure is the segmented LH value obtained from CBS. It has two regions. One which corresponds to the normal zone (CN = 2) and the other to both deleted and amplified regions. The algorithm does not distinguish between the deleted and the amplified zone. This fact is not a concern since it differentiates the normal regions from the aberrated ones, which is the objective here.

Figure 1 displays the CNs, the fraction of B-allele (*β*) and the **LH** of chromosome 8 in sample GSM318736, from the Prostate Cancer experiment. The top panel shows three different zones in this sample: a normal zone in the beginning of the chromosome (CN=2, from 0 to 20 Mb), a deletion in the zone of the *p* arm closer to the centromere (CN=1, from 20 to 45 Mb) and a gain in the *q* arm of the chromosome (CN=3, from 45 to 147Mb). These regions can be inferred from the CN plot, but also from the *β* plot (middle panel). On the other hand, in the bottom panel, the **LH **plot shows two clouds in the normal region: one is centered at 0 and the other at 1. The distribution is also bimodal for 3 copies, but the peaks for the distributions are, in this case, about 0 and 0.7. The density function of **LH** in a deletion presents a plateau for low values (close to 0), and it is unimodal if there is no normal contamination.

HNCNs have a distribution that peaks at **LH**=1 (the place where *θ*_*A *_=* θ*_*B*_). The density function of **LH** for homozygous SNPs has a peak close to 0 (row two in Table 1). The specific position of the peak depends on the summarization method, especially on how well the method deals with cross-hybridization between probes that measure different alleles. The expected values of **LH** for the SNPs with CN different to 2 are shown in Table 1 (The last genotype marked with an asterisk, implies a simultaneous alteration in both chromosomes which is very unlikely to occur).

**Table 1:** Level of Heterozygisity

**Genotype**	**CN**	**LH**
-	0	NAN
A,B	1	0
AB	2	1
AA,BB	2	0
AAB,ABB	3	2/3
AAA,BBB	3	0
AAAB,ABBB	4	1/2
AAAA,BBBB	4	0
AABB	4	1^∗^

#### Selecting Heterozygous SNPs with Neutral CNs (HNCNs)

Using Table 1, a threshold *LH*^*th *^has been selected to discern whether the corresponding SNP is HNCN or not. The most critical case to discern from a normal region (using the **LH** value) is a zone with 3 copies. The expected **LH** value is around 2/3 for a perfect summarization model with a pure tumor sample. If there is contamination from the surrounding normal tissue, this value will be larger. Because of this, a suitable threshold is 5/6 (the middle point between the expected value for normal heterozygous calls and the value for 3 copies).

Once the threshold is set, the SNPs with **LH** value over it are labeled as HNCN (**LH **= 1) and the others as non-HNCNs (**LH **= 0). A segmentation algorithm is applied to these binary data to find zones enriched in HNCNs. We used a variant of CBS in which the input are binary data [18,19], although other methods could be applied (using for example Hidden Markov Models [20]).

In the aforementioned Figure 1, the thick gray lines in the bottom panel represent the different segments predicted by CBS. The *y* axis represents the proportion of HNCNs SNPs in the segment. It can be observed that CBS detects 2 different segments, corresponding to the two states (normal and aberrated). In other samples, segmentation also differentiates between aberrations (deletions and gains) assigning them a different value since the proportion of SNPs whose **LH** is above the threshold can be different, but this fact does not affect the method.

#### Labeling normal segments

On average, 27% of the SNPs in a HapMap sample are heterozygous. Therefore, ideally, the proportion of HNCNs in a normal region would be around 27%. On the other hand, the proportion of HNCNs should be ideally close to 0% in an aberrated region. We have selected a threshold in the middle point (13.5%). The larger the threshold, the more likely the selected segments have neutral CNs. However, some normal regions can be missed because of noise or simply because the regions include many SNPs with one rare variant.

For example, consider the initial part of the *p* arm from Figure 1, which is normal, and the *q* arm, which is amplified. The density function for the normal zone has more SNPs with LH closer to 1 than the amplified region. In this particular case, 18.3% of the SNPs are above the threshold set in the previous section. For the 3 copies region, only 8.6% of the SNPs have LH above the threshold. The difference between both regions is large enough, so that, the algorithm is not sensitive to a particular selection of the threshold.

If the array of the experiment includes SNP and CN probes (GWS 5.0 or GWS 6.0), then the corresponding status (neutral copy number or not) of the CN probes is inferred from the status of the segment where they are located (that has been computed using the SNP probes).

### Copy number data scaling

The final processing of NSA includes two scaling steps, one by SNPs and another by samples.

#### Scaling by SNP

NSA implements two methods (user-selectable) to compute the references: the first one uses standard medians and the second one uses weighted medians to minimize batch effects. In this second method each sample has a different computed reference.

For the first method, the reference is computed by using the median of the signals of the samples labeled as normal for each SNP. In the second case, a different algorithm (described in the following section) estimates some weights that are used to compute the reference for each SNP and sample using weighted medians [21]. It also uses only the samples labeled as normal for each SNP to compute the reference.

#### Scaling by sample

The algorithm computes for each sample the median of the CNs of the SNPs assumed to be normal and re-scales all the data so that the median of the normal zones for each sample is 2.

The two scaling steps of the proposed normalization is similar to a median polish in which only the SNPs which are labeled as normals are included in the computation.

#### Algorithm

The procedure that involves both scaling steps is the following: 

1. Get the reference signal for every SNP computing:

(3) $${\text{Ref}}_{j}^{\text{SNP}}={\text{median}}_{i\in I({\text{SLH}}_{j,i})}(\theta_{j,i}^{\left(1\right)})$$

where $\theta_{j,i}^{\left(1\right)}=\theta_{j,i}^{A}+\theta_{j,i}^{B}$ is the sum of the signals of both alleles and *I*(*SLH*_*j*,*i*_) is an indicial matrix of the samples (for SNP *j*) that have been labeled as normal in that position. *Ref*_*j*_^*SNP*^ is the computed reference for SNP *j*, taking the medians by SNPs. If the batch effect removal (BER) method is used, this step converts into

(4) $${\mathrm{Ref}}_{j,i}^{\text{SNP}}={\text{wmedian}}_{k\in I({\text{SLH}}_{j,k})}(\theta_{j,k}^{\left(1\right)},\gamma_{i,k}).$$

where *wmedian* stands for the weighted median using weights *γ*_*i*,*k*_. Notice that the reference is different for each SNP *j* but also for the same SNP in a different sample *i*, since the weights of the median (*γ*_*i*,*k*_) are different for each sample. These weights are computed by the algorithm described in the following section.

2. Normalization of the signals across SNPs. For each SNP in every sample

(5) $$\theta_{j,i}^{\left(2\right)}=\frac{2\theta_{j,i}^{\left(1\right)}}{{\text{Ref}}_{j(,i)}^{\text{SNP}}}$$

The values of $\theta_{j,i}^{\left(2\right)}$ will be close to the real CNs. The reference will be different for each sample if the batch effect removal method is used.

3. Get the reference signals for every sample:

(6) $${\text{Ref}}_{i}^{\text{sample}}={\text{median}}_{j\in I({\text{SLH}}_{j,i})}(\theta_{j,i}^{\left(2\right)})$$

*Ref*_*i*_^*sample*^ is the average value (using medians) of the normal segments of the genome. This value is expected to be close to 2. In order to ensure this, the following (and last) step scales the samples accordingly.

4. Normalization of the signals across the samples. For each sample, in every SNP

(7) $$\theta_{j,i}^{\left(3\right)}=\frac{2\theta_{j,i}^{\left(2\right)}}{{\text{Ref}}_{i}^{\text{sample}}}$$

The values of $\theta_{j,i}^{\left(3\right)}$ are the final estimates of NSA for the total CN values.

These steps should be repeated until convergence but improvement is negligible after the first iteration (average copy number changed about 0.001 copies in the examples).

### Computation of weights for batch effect removal

Batch effects have proved to have paramount importance in the analysis of SNP arrays [22,23]. The particular characteristics of NSA algorithm helps to develop an algorithm to minimize them. The overall idea is that, since NSA identifies normal zones that are expected to have neutral CNs, the reference can be selected so that, for these normal zones, the estimated copy number is close to 2.

The procedure is the following. First of all, a set “*S*” of SNPs is selected. This set must include a sufficient number of normal SNPs to capture the relationships between the arrays. These SNPs are selected from normal regions in most of the samples. Although for some studies there are no normal SNPs for all the samples, the SNPs in “*S*” are selected so that they appear as normal in as many samples as possible.

For SNPs in “*S*” that are not located in normal regions in any of the samples, their values are substituted by the references using standard medians, the signal used by the algorithm $\hat{\theta }$ is

(8) $${\hat{\theta }}_{j\in S,i}=\left\{\begin{array}{c} \theta_{j\in S,i}^{\left(1\right)},\text{if}i,j\in I(\text{SL}H_{j,i})\\ {\text{Ref}}_{j\in S}^{\text{SNP}},\text{otherwise} \end{array}\right)$$

Using these values, the weights *γ*_*i*,*k *_to compute the reference for each sample *i* are estimated by solving the following optimization problem

(9) $$\underset{\gamma_{i,k}}{\text{min}}||\text{log}({\hat{\theta }}_{j\in S,i})-\overset{I}{\underset{k=1,k\neq i}{\sum }}\gamma_{i,k}\text{log}({\hat{\theta }}_{j\in S,k})||$$

subject to

(10) $$\gamma_{i,k}>0,\gamma_{i,i}=0$$

implicitly assuming that the reference for $\text{log}(\hat{\theta })$ is $\sum \gamma_{i,k}\text{log}({\hat{\theta }}_{j\in S,k})$, i.e. a linear combination of the logarithm of the signals for each sample. The restrictions impose that 1) sample *i* is not used to compute its own reference and 2) only positive weights are allowed, i.e. if samples *i* and *k* are so different that the corresponding coefficient *γ*_*i*,*k*_ is negative, sample *k* is not used to build the reference of sample *i* instead of giving it a counter-intuitive negative weight.

This optimization is a quadratic programming (QP) problem. Instead of using a standard QP algorithm (quite time consuming) we have iteratively solved the minimum squares problem. In each step, the algorithm removes the samples whose weights are negative in the solution.

Any linear combination such as $\sum \gamma_{i,k}\text{log}({\hat{\theta }}_{j\in S,k})$, can be interpreted as a weighted mean multiplied by an additional factor (the sum of the weights). In this particular case, since the data are further normalized by samples, this additional factor is computed and taken into account in the “scale by samples” step. In addition, instead of using the weighted mean, we used a weighted median to increase the robustness and withstand the presence of outliers. Since the median and the logarithm are interchangeable operators, the suggested reference is

(11) $${\text{Ref}}_{j,i}^{\text{SNP}}={\text{wmedian}}_{k\in I({\text{SLH}}_{j,k})}(\theta_{j,k}^{\left(1\right)},\gamma_{i,k}).$$

Using this formula, each of the computed *γ*_*i*,*k *_is the weight of the normal regions of sample *k* to compute reference for sample *i* using a weighted median. For each SNP *j*, only the samples that are expected to be normal are included in calculation of the median using the corresponding weights.

## Results

In this part, it is shown that the results using NSA outperforms the use of control samples from a different lab or using a robust median of the tumoral samples (which are the most used methods). This improvement in performance appears both in noise and bias. Since there is not a ground truth to compare against, we have used three indirect aspects to state the performance: the ability to find CNAs along the genome, the quality of the estimated CNs in regions that are known to be normal and the ability to find recurrently aberrated regions.

Eckel-Passow *et al.*[24] shows a comparison among different summarization algorithms. It describes four summarization methods [2-5]. Within these methods, there are only two different algorithms for scaling: using the median (of some of the samples) and a linear model. The summarization methods used here (ACNE and CalMaTe), internally implement a callibration that is in fact a linear model. In addition to the methods described in [24], dChip [6] implements a trimmed mean of the samples if no references are provided, LaFramboise [25] fits a non-linear model using the information from control samples, Nannya *et al*[26] propose to use the m control samples most similar to the sample under study. In the end, the suggested methods to scale the samples (except [26]) are simply the computation of a robust average (i.e. the median or a trimmed mean) of some of the samples. As will be shown later, our approach to remove batch effects resembles Nannya’s but focusing only on the normal zones within the tumor samples.

We have compared four different possibilities to select the samples to build the references. These scaling methods depend on whether control samples are available or not and on the laboratory where the control samples (if any) were hybridized. The first algorithm under study can be applied to a dataset which includes control samples from the same lab. In this case, for each SNP, the median of the values of the control samples is used as reference i.e, the median of ( $\theta_{C,i}^{A}$ + $\theta_{C,i}^{B}$) where *C* is a subset of control samples within the experiment. We named this algorithm MCS (median of control samples). This is the method of choice suggested by all the referred methods if control samples are available.

The next three algorithms are used when no control samples are available. The second method (MHS: median of HapMap samples) computes the reference using the median of external control samples (in this case they are from HapMap). This is the method suggested by [3,6]. The third method (MTS: median of tumoral samples) builds the reference using the median of all the tumoral samples (implicitly assuming that most of the samples have neutral copy number at a given locus). It is suggested in some vignettes of the package aroma.affymetrix [2]. And, finally NSA, which calculates the reference based on the predicted normal regions within the tumoral samples.

### Ability to find recurrent aberrant regions

We have focused this section on the detection of recurrent alterations since it is the aim of many CN analysis.

The analyzed experiment is the GBM dataset (64 *tumoral* samples [8] hybridized to Affymetrix Mapping50K_240Xba). The data were analyzed using CRMAv2 pre-processing and ACNE summarization method. Once the *θ* values were obtained, the data were scaled using both MTS and NSA. In addition, we performed the same analysis taking HapMap samples as references (MHS).

After applying the three scaling methods, the CN estimates were segmented using CBS [18] and then, the recurrent aberrant regions were calculated using GISTIC [27].

**Figure 2:**
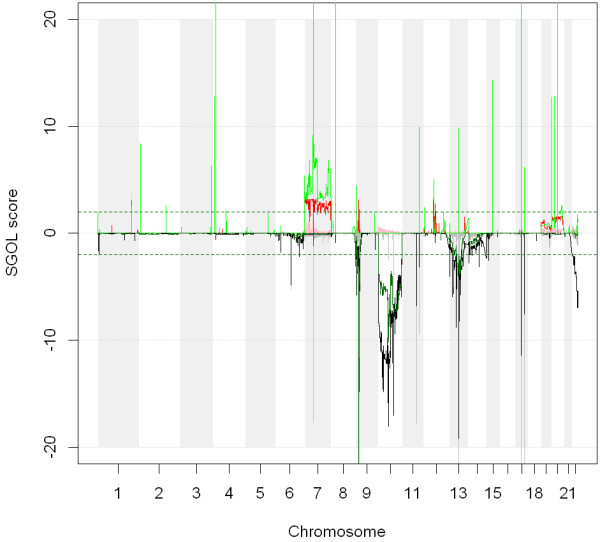
**This figure shows the recurrent aberrations obtained after using MTS, MHS and NSA scaling methods.** MTS is represented with thick red and grey, MHS with green and darkgreen lines and finally, NSA with red and black lines. The performance for MHS and NSA in terms of identifying recurrent aberrations is similar. NSA and MHS provide more significant values for the recurrent aberrations and no artificial aberrations appear. On the contrary, MTS introduces artificial aberrations.

GBM has been deeply studied by different groups [8,28], and it is known to present strongly recurrent aberrations. For example, in GBM there is a well known recurrent amplification in 7*q* and a deletion of almost the whole chromosome 10 [28]. Figure 2 shows the recurrent aberrations found using the median of the tumoral samples (MTS), shown in thick red and grey, the ones obtained from NSA (lines red and black) and MHS (green and dark green). Using MTS, the aberrated region 7*q* does not only appear to be amplified but also deleted. This recurrent deletion is an artificial aberration originated by the bias of the estimation of the reference. Since the recurrent amplification in 7*q* occurs in more than half of the samples, the MTS estimates of the reference for these loci are larger than the real ones. In turn, the scaling process provides smaller copy number values than expected for these loci. This is a general trend: in recurrent amplifications, MTS estimates are larger for neutral copy number loci than expected. This bias makes the amplification less prominent and the statistics less significant. The opposite effect can also be seen in chromosome 10, which is known to be recurrently deleted. Using MTS, both a recurrent amplification and a recurrent deletion appear in chromosome 10.

On the other hand, in both chromosomes 7*q* and 10, NSA and MHS provide more significant values for the aberrations (the amplified and deleted regions have higher q-values) and no artificial aberrations appear. The performance in terms of identifying recurrent aberrations is similar for both algorithms. Nevertheless, we have to fine-tune the low-level analysis using MHS since a very strong bias appears in the CN estimates (that NSA does not present because of the second stage of the algorithm -scale by samples).

### Ability to find CNAs along the genome

We have performed this comparison in two cancer datasets, Prostate Cancer and Lung Cancer. In addition, we have analyzed the chromosome X in some HapMap samples to check if NSA is able to uncover which samples have two copies (female samples) and, using the first autosomic region of this chromosome, compare the ability to find a copy number change.

#### Prostate cancer analysis

The Prostate Cancer dataset is hybridized to Affymetrix Mapping250K_Nsp [9]. We compared the NSA results with MTS, MCS and MHS. It is reminded that MCS needs more hybridizations than the other methods. This analysis is focused on finding aberrant regions in order to show which method detects CNAs more accurately.

**Figure 3:**
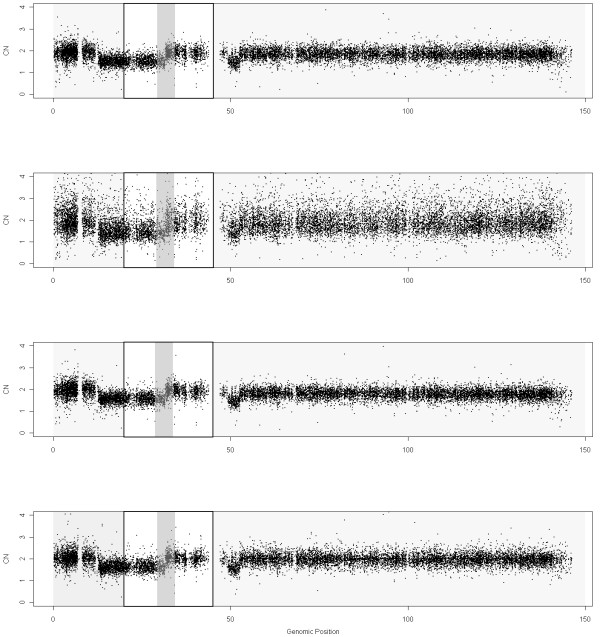
**DNA copy numbers using MCS (first panel, reference generated using control samples from the sample lab, 40 samples in total), MHS (second panel, reference generated using Hapmap samples, 40 samples in total), MTS (third panel, reference generated using all tumoral samples (20 samples)) and NSA (fourth panel, reference using “normal” zones within tumoral samples (20 samples)) for chromosome 8 in sample GSM318766 from the Prostate Cancer dataset hybridized to Mapping250K_Nsp.** Noise is especially large for MHS method.

Figure 3 presents the DNA CNs of chromosome 8 for sample GSM318766 obtained using the different scaling methods. It is not possible to pinpoint clear differences from these figures, except for the MHS method which is clearly noisier than the others in this example.

**Figure 4:**
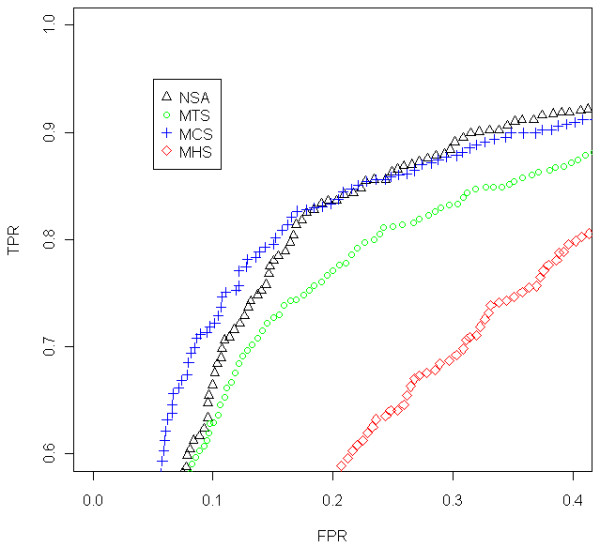
**ROC comparison with scaled data (Prostate Cancer dataset, sample GSM318766, hybridized to Mapping250K_Nsp).** We have focused on the region where there is a change from 1 to 2 copies, around position 32Mb, that appears in Figure 3. For the analysis, we have included the SNPs that surrounds the copy number change (specifically from 20 to 45 Mb). The SNPs within a safety zone from 30Mb to 34 Mb are not considered in the analysis, because it is difficult to discern the exact position of the change. The SNPs located upstream the change point are considered to have total CNs equal to 1 (deleted region) and the SNPs downstream the change point to have a normal CN (CN=2). For this particular sample (in the studied jump) the results using NSA are almost as good as using MCS. MTS gives poorer results and MHS is well behind. This is a general trend that discourage the joint analysis of samples from different laboratories.

In order to quantify the ability to find CNAs we have generated a ROC curve (Figure 4) for the region that changes from 1 to 2 copies, around position 32Mb (Figure 3). The SNPs located downstream the change point are considered to have a larger total CN (which have neutral CNs) than the SNPs upstream the change point (deleted region). For any threshold in the CNs there are true positives TP (SNPs in the normal region that are above the threshold), false positives FP (SNPs in the deleted region that are above the threshold), true negatives TN (SNPs in the deleted region below the threshold) and false negatives FN (SNPs in the normal region that are below the threshold). The FPR = FP /(FP + TN) and TPR = TP /(TP + FN) can be evaluated at different thresholds. The ROC curve is the plot of TPR against FPR for different thresholds. A perfect classification method provides large TPRs for low FPRs. The worst classification method (random selection) is a straight line from (0,0) to (1,1). This evaluation method was also used by Bengtsson et al. in [1] and it is deeply explained in their corresponding Supplementary Notes.

For this analysis, we have included the SNPs that surround the CN change (specifically from 20 to 45Mb). Since the exact position of the change is difficult to locate, the SNPs within a safety zone from 30Mb to 34Mb are not considered. This figure includes some gray zones that illustrate which is the region under study. It can be seen in Figure 4 that MCS and NSA are the ones that best identify this CN change (better TPR for the same FPR). MTS gives intermediate results and MHS performs worse than the rest. We have selected this locus since it is the most prominent recurrent aberration for this dataset and has been previously shown to be a frequent aberration in Prostate Cancer [29,30].

#### Lung cancer analysis

We have also validated NSA with the dataset from a Lung Cancer study [10], hybridized to Affymetrix GenomeWideSnp 6.0. It should be noted that in this study there are 291 samples where 59 are control samples from a different lab (one of the authors informed us in a personal communication that they are from HapMap).

**Figure 5:**
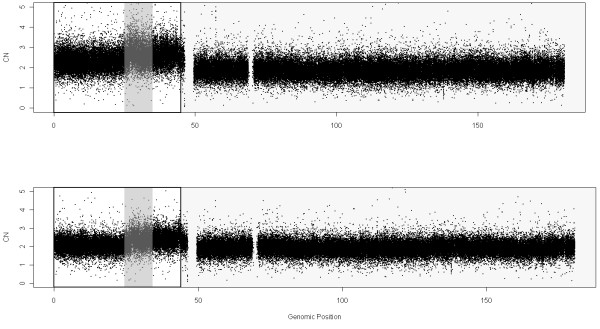
DNA copy numbers using MHS (291 samples where 59 are control samples from HapMap, used to calculate the reference) and NSA (reference using “normal” zones within tumoral samples (232 samples)) for chromosome 5 in sample GSM638958 from the Lung Cancer dataset hybridized to GenomeWideSNP 6.0.

**Figure 6:**
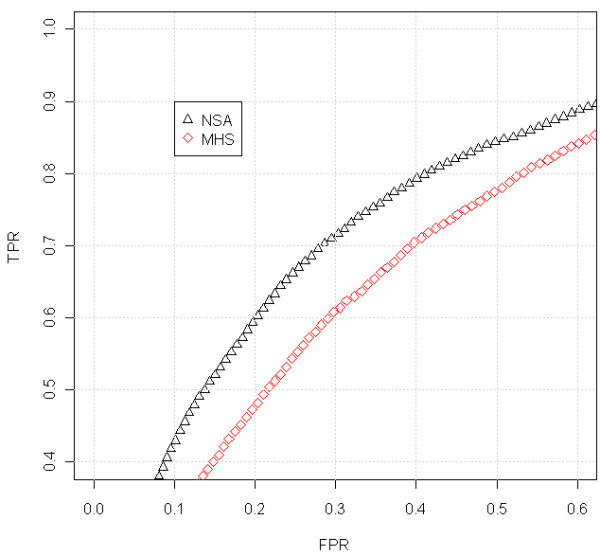
**ROC comparison with scaled data (Lung Cancer dataset, sample GSM638958, hybridized to GenomeWideSNP 6.0 array).** We have used the region where there is a change from 2 to 3 copies, around position 30Mb, that appears in Figure 5. For the analysis, we have included the SNPs that surrounds the copy number change (specifically from 0 to 45 Mb). The SNPs within a safety zone from 25Mb to 35 Mb are not considered in the analysis because it is difficult to discern the exact position of the change. The SNPs located upstream the change point are considered to have total CNs smaller than the SNPs downstream the change point. For this particular sample (in the studied jump), NSA differentiates the two regions better than MHS.

**Figure 7:**
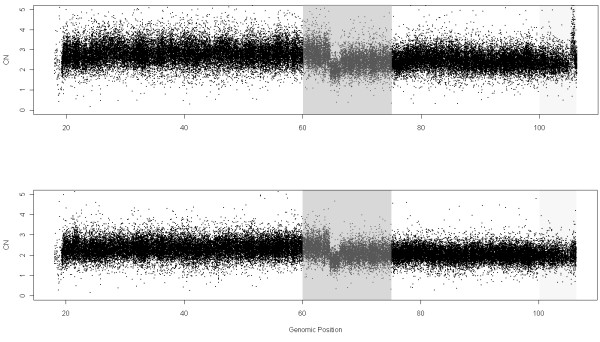
DNA copy numbers using MHS (291 samples where 59 are control samples from HapMap, which are the ones used to calculate the reference) and NSA (reference using “normal” zones within tumoral samples (232 samples)) for chromosome 14 in sample GSM639066 from the Lung Cancer dataset hybridized to GenomeWideSNP 6.0.

**Figure 8:**
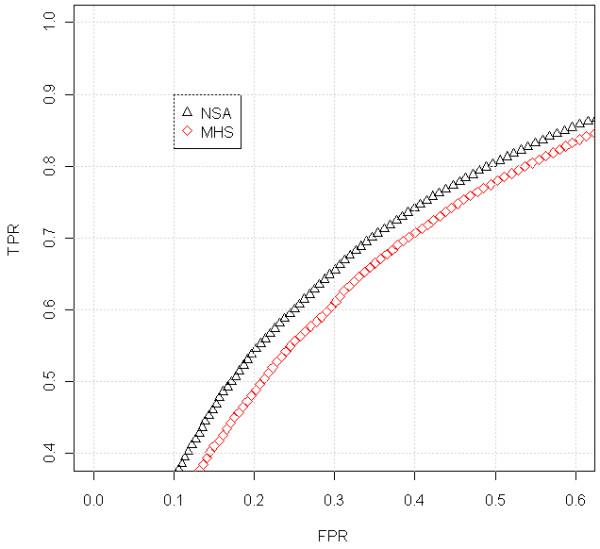
**ROC comparison with scaled data (Lung Cancer dataset, sample GSM639066, hybridized to GenomeWideSNP 6.0 array).** We have used the region where there is a change from 3 to 2 copies, around position 65Mb, that appears in Figure 7. For the analysis, we have included the SNPs that surrounds the copy number change (specifically from 0 to 100 Mb). The SNPs within a safety zone from 60 Mb to 75 Mb are not considered in the analysis since includes a deleted region. The SNPs located upstream the change point are considered to have total CNs larger than the SNPs downstream the change point. For this particular sample (in the studied jump), NSA differentiates better than MHS between the two regions.

Figure 5 shows the CNs of chromosome 5 in sample GSM638958 using MHS (over the 291 samples) and NSA (using the 232 tumoral samples). It can be seen from this figure that the noise using MHS is again larger than using NSA. The region used here to calculate the ROC ranges from 0 to 45 Mb and the safety region is from 25 to 35 Mb where there is a jump from 2 to 3 copies [31,32]. The ROC curve from Figure 6 shows that NSA performs better than MHS. Figures 7 and 8 shows a similar behavior in a different sample and location.

#### Chromosome X analysis

**Figure 9:**
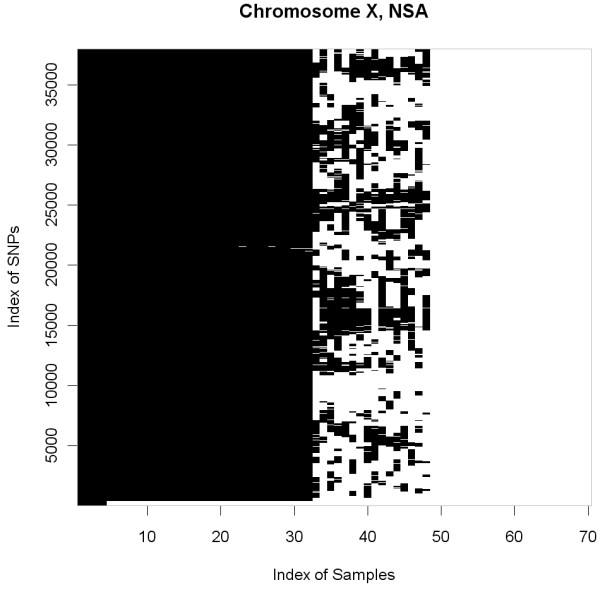
**This figure shows the normal regions (in white) detected by NSA within the chromosome X from the GIGAS HapMap dataset.** These data (70 samples hybridized to GenomeWideSNP 6.0 array) were preprocessed with ACNE. Note that the index of SNPs is ordered by genomic position and the index of samples is ordered by “normal” content. According to this order, the first 32 samples are men and the next 38 are women. In addition, female samples can be separated into two groups. The first group, that presents many uniparental disomy regions, belongs to Asian women (except one caucasian) and the second group belongs to caucasian and Yoruban women.

Although NSA is not thought to be used on sexual chromosomes, we have included an analysis of chromosome X in a set of HapMap samples that includes 32 male and 38 female samples. This set of samples is equivalent to an experiment in which some of the samples have a deletion of one of the chromosomes. Figure 9 depicts which regions are identified as normals (in this case should correspond to female samples) by NSA. The first 32 samples are males and the last 38 samples are females. Within the male samples, the beginning of the X chromosome PAR1, one of its autosomic regions- is correctly identified to have two copies. The second autosomic region (PAR2) (a few SNPs at the end of the X chromosome) also appears with two copies. The XTR (X-transposed region, around the middle of the q arm) showed very few HNCNs and did not pass the threshold for almost all the samples. Among the female samples we found that a group of them (from sample 33 to sample 48) presented many regions with uniparental disomy i.e. zones with LOH. All these samples were from Asian women (and one Caucasian). The second group were all Caucasian and Yoruban, this group did not show LOH regions and were predicted to be normal for the whole chromosome.

**Figure 10:**
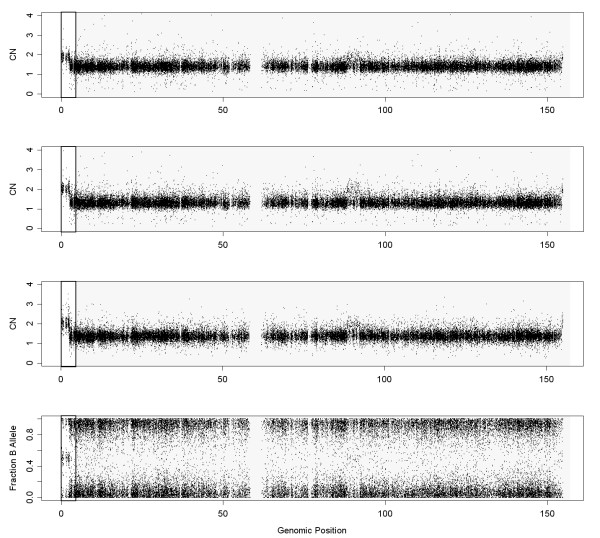
**These figures show the DNA copy numbers using ACNE, ACNE+NSA and CN5 and the B Allele Fraction for chromosome X in sample GIGAS_g_GAINmixHapMapAffy2_GenomeWideEx_6_A04_31266 from the GIGAS HapMap dataset hybridized to GenomeWideSNP 6.0.** The number of copies and the fraction of the B allele show that this sample is male. The total copy number for the autosomal regions -beginning of p arm and middle of q arm- are closer to two copies.

**Figure 11:**
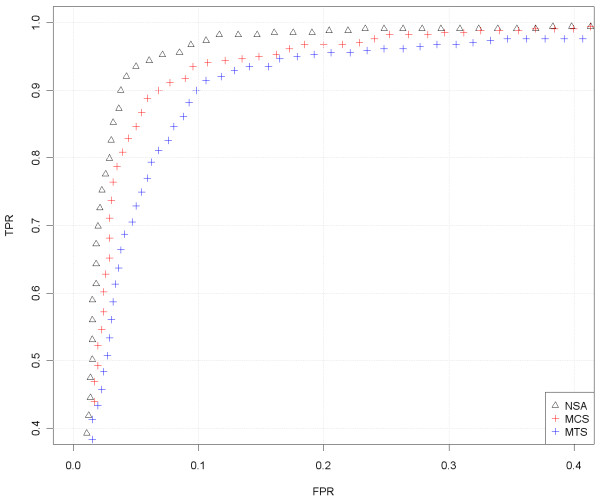
**ROC comparison with scaled data (GIGAS HapMap dataset, sample GIGAS_g_GAINmixHapMapAffy2_GenomeWideEx_6_A04_31266, hybridized to GenomeWideSNP 6.0 array).** We have used the initial region of chromosome X, where there is a change from 2 to 1 copy, that appears in Figure 10. For the analysis, we have included the SNPs that surrounds the copy number change (specifically the first 1000 SNPs, ordered by genomic position). There is no safety zone in the analysis. The SNPs located upstream the change point are considered to have total CNs larger than the SNPs downstream the change point. Again, for this particular sample (in the studied jump), NSA differentiates better than ACNE (in this case, “MTS”) and CN5 (in this case, “MCS”).

Using this information, we have compared ACNE, ACNE+NSA with CN5 (see Figure 10). CN5 uses the information of sexual chromosomes (i.e. which samples are male and female) to perform the scaling. In this particular analysis, ACNE and NSA do not have this information. Plain ACNE can be considered to scale by using the MTS method (all the samples are used as reference regardless of the sex) and CN5 by using MCS (uses the expected number of copies for female samples). We have analyzed the CN jump located at the end of the PAR1 region of chromosome X in a male sample. This pseudoautosomal region goes from 0.6 Mb to 2.699 Mb. The ROC curve in Figure 11 shows that, ACNE performs worse than CN5 in this particular analysis. However, after correcting ACNE with NSA, its ROC outperforms CN5’s.

### CN estimates for normal regions

**Figure 12:**
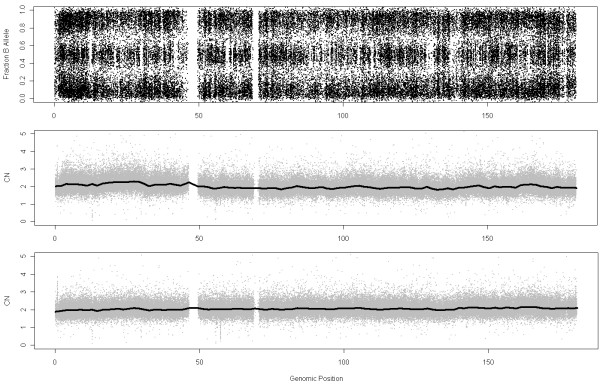
**These figures show Fraction of B allele and DNA copy numbers using MHS (291 samples where 59 are control samples from HapMap, which are the ones used to calculate the reference) and NSA (reference using “normal” zones within tumoral samples (232 samples)) for chromosome 5 in sample GSM638955 from the Lung Cancer dataset hybridized to GenomeWideSNP 6.0.** FracB plot shows that the sample has neutral copy number. MHS predictions show a wavy effect that is not present in the case of NSA.

In the previous paragraphs we have shown that using samples from different labs increases the noise of the estimates. However, it will be shown here that this fact does not only increase the noise but also the bias. We have used again the Lung Cancer dataset hybridized to Affymetrix GenomeWideSNP 6.0 array and chosen a chromosome in a tumoral sample which seems to be normal (since the fracB plot shows three clouds through the whole chromosome, Figure 12). These three clouds correspond to SNPs with AA, BB and AB genotypes. Figure 12 shows the CN estimates using MHS (middle panel) and NSA (bottom panel). A wavy effect on the whole chromosome appears when using MHS, which does not occurs using NSA. This bias that changes along the genome is originated by a wrong computation of the reference.

Moreover, there is also another bias, which exists even using control samples from the same lab. This bias is generated in the pre-processing step where the samples are normalized. This step performs a transformation on probe level data to make them comparable across different samples. This transformation can be a quantile scaling or simply a multiplication of the data by a constant. Nevertheless, if a large part of the genome in a sample is deleted (amplified), the normalization procedure tends to compensate for this effect and the normal regions of the genome (that ideally have CN=2) are shifted to a larger (smaller) value to compensate for the aberration. This fact induces a bias in the CN estimation. Therefore, if there is a sample with many deletions (or amplifications), the normal regions of these samples have a slightly higher (lower) value than 2.

### Behavior in the Presence of Batch Effects

We have also analyzed the ability to remove batch effects using NSA. For this we have used the Ovarian Cancer dataset [11]. This dataset includes 129 samples (72 tumoral and 57 references, some of them matched) that were hybridized in 13 batches. The number of samples for each of the batches is very different to each other ranging from 2 samples in one batch to more than 20 samples in another batch. In the case of small batches, the advantage of computing the references by batches or taking the experiment as a whole is unclear.

We have applied NSA with batch effect removal on this dataset. NSA’s procedure to estimate the weights is completely blind, i.e., it does not require any information from the user on which are the samples within each batch.

**Figure 13:**
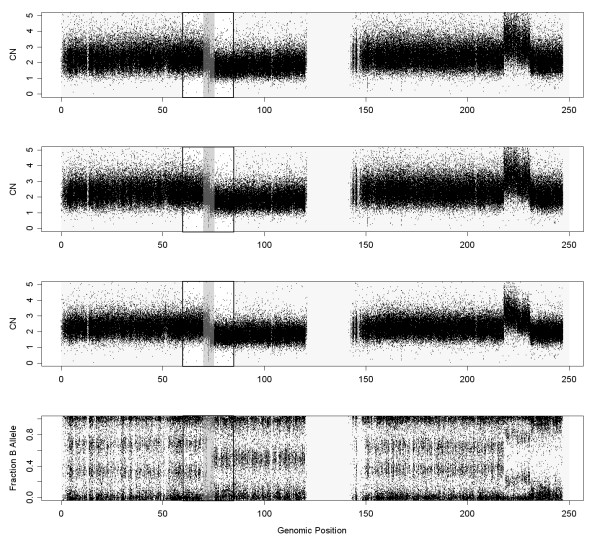
**These figures show the DNA copy numbers using CalMaTe, CalMaTe+NSA (without BER) and CalMaTe+NSA (with BER) and the B Allele Fraction for chromosome 1 in sample GSM492511_IC318T from the Ovarian dataset hybridized to GenomeWideSNP 6.0.** It can be observed that the resultant signal obtained by NSA with BER is less noisy than the other methods.

**Figure 14:**
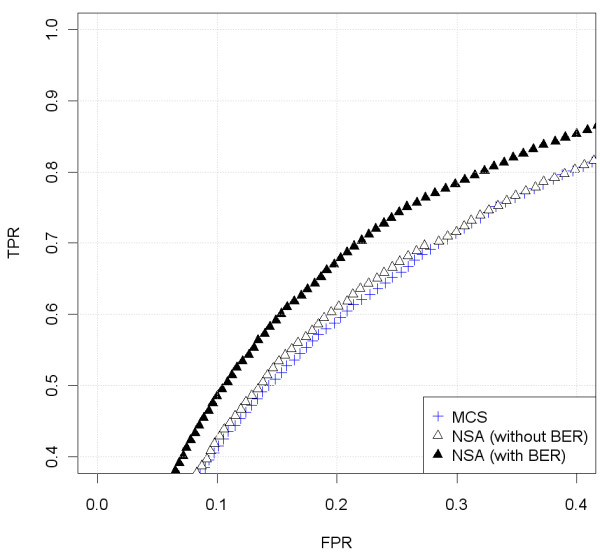
**ROC comparison with scaled data (Ovarian dataset, sample GSM492511_IC318T, hybridized to GenomeWideSNP 6.0 array).** We have used the region where there is a change from 3 to 2 copies, in the begining of chromosome 1, that appears in Figure 13. For the analysis, we have included the SNPs that surrounds the copy number change (specifically from 60 to 85 Mb). The SNPs within a safety zone from 70 Mb to 75 Mb are not considered in the analysis. The SNPs located upstream the change point are considered to have total CNs larger than the SNPs downstream the change point. For this particular sample (in the studied jump), NSA (with BER) differentiates better than NSA (without BER) and CalMaTe using control samples as references (MCS).

**Figure 15:**
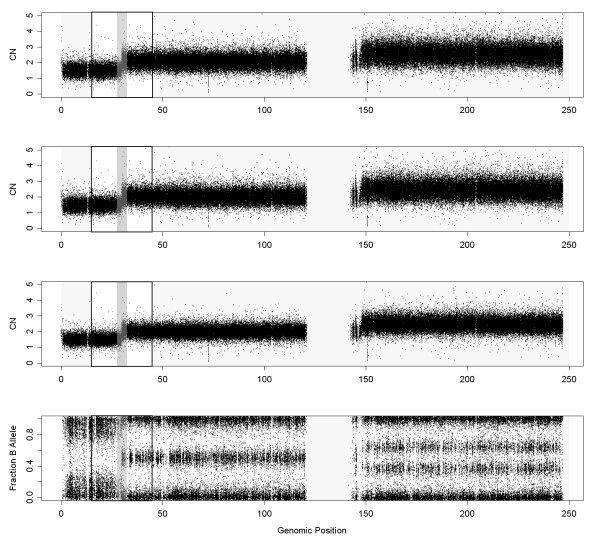
**These figures show the DNA copy numbers using CalMaTe, CalMaTe+NSA (without BER) and CalMaTe+NSA (with BER) and the B Allele Fraction for chromosome 1 in sample GSM492507_IC288T from the Ovarian dataset hybridized to GenomeWideSNP 6.0.** The resultant signal obtained by NSA (with BER) is less noisy than using other methods.

**Figure 16:**
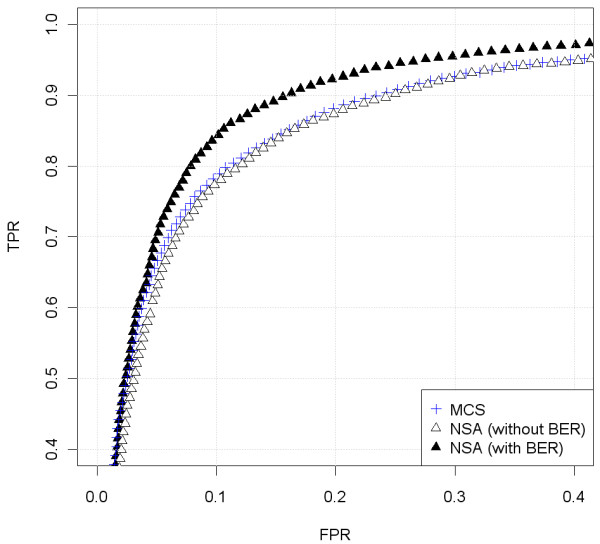
**ROC comparison with scaled data (Ovarian dataset, sample GSM492511_IC318T, hybridized to GenomeWideSNP 6.0 array).** We have used the region where there is a change from 1 to 2 copies, in the begining of chromosome 1, that appears in Figure 15. For the analysis, we have included the SNPs that surrounds the copy number change (specifically from 15 to 45 Mb). The SNPs within a safety zone from 27.5 Mb to 32.5 Mb are not considered in the analysis. The SNPs located upstream the change point are considered to have total CNs smaller than the SNPs downstream the change point. NSA (with BER) outperforms NSA (without BER) and CalMaTe.

Figure 13 shows the chromosome 1 in sample GSM492511_ICT318T using CRMAv2 plus CalMaTe summarization method and NSA to scale the result. First panel shows CalMaTe using the control samples as references (taking all of them as a whole) and second and third panel show the scaling after running NSA without and with Batch Effect Removal (BER). It can be seen that the overall noise is slightly smaller using NSA with BER. To quantify this effect, we have also included the ROC curve (Figure 14) of the CN change around position 70 Mb. This ROC confirms what is seen by bare eye: the noise is smaller and therefore, the ability to detect copy number changes improves. We have included also the analysis in a different sample (GSM492507_IC288T) of a change from 1 to 2 copies (Figure 15). This general trend is similar. This copy number change is easier to pinpoint as shown by the upper location of the ROC curve (Figure 16). The behavior of NSA (without BER) and MCS is similar. Therefore, NSA can be used safely without the need to provide the control samples to the scaling method.

**Figure 17:**
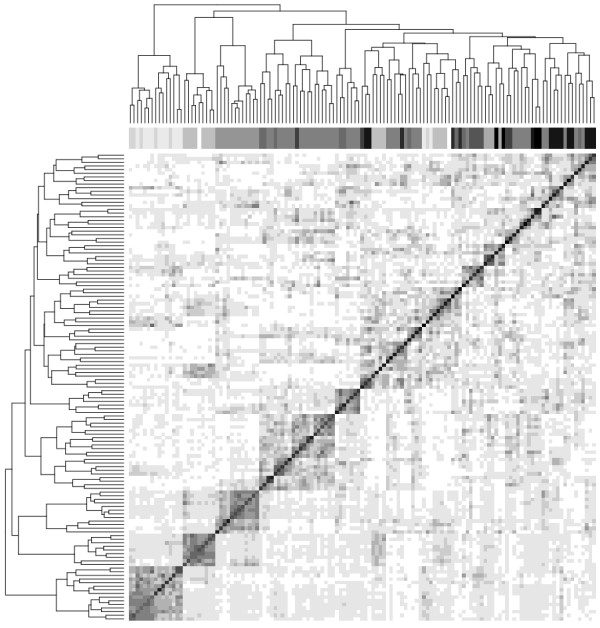
**Hierarchical clustering performed on the correlation of the weighting matrix (*γ***_***i***,***k***_**) calculated for the Ovarian dataset.** This dataset have been run in 13 batches. Each batch has a different color (gray scale). The assigned gray scale is done according to date of hybridization. A gray scale band is added in top of the figure. It can be observed that the weights are similar for samples hybridized in similar batches.

Figure 17 shows a hierarchical clustering of the correlation of the matrix of weights *γ*_*i*,*k*_. It can be seen that the weights are similar for samples hybridized within the same batch. Each batch have a different color (gray scale). The shades of gray are ordered by hybridization day.

## Discussion and conclusions

This paper describes NSA, an algorithm to scale the summarized SNP signals to CN values by finding normal regions within tumoral samples. NSA is platform (Illumina or Affymetrix) and pre-processing method (dChip, CRMAv2, ACNE, CalMaTe…) independent. The synthetic reference generated by NSA using only tumoral samples gives more accurate results than either using control samples from different labs or using all the tumoral samples. Indeed, NSA results are close to the ones obtained using control samples from the same lab within the dataset. In addition, NSA includes an algorithm to deal with batch effects. It automatically computes an optimal reference for each sample (that in our tests is strongly related to the hybridization batches). Batch information is not required to run NSA; the algorithm automatically identifies the proper samples to compute the reference using only the signals of the microarrays. NSA minimizes the problem of bias for samples with a large number of similar aberrations (i.e. most of them are gains or deletions). For these samples, the predicted CNs for normal regions tends to compensate the aberration including a bias. This is a potential problem that also occurs using MCS (control samples from the same lab). NSA is able to effectively discover the normal regions and uses them to scale the data diminishing any bias that appears in the normalization step.

We have compared NSA with other scaling methods. On one hand, it is important to pinpoint that the number of hybridized samples needed for NSA, MTS and MHS is much smaller than using MCS, since these methods do not need to hybridize control samples from the same lab to create the reference. MHS method seems to be noisier which can be an effect of the difference between the protocols or conditions of the labs where the arrays were hybridized. MTS provides biased estimates especially in the case of recurrent aberrations. On the contrary, NSA estimates are similar to the ones from MCS. In addition, NSA provides a way to deal with the unavoidable batch effects of large experiments.

NSA also presents some limitations that are summarized here. Ultimately, the identification of normal regions relies on the fact that gains in both chromosomes are very unlikely to occur. If this is not the case and a few samples do present amplifications on both chromosomes, NSA is still reliable since a robust estimator of the reference (such as the median) can withstand the presence of a small percentage of outliers.

The prediction of normal regions can be affected by samples with low tumor purity that can be wrongly included within the normal ones. Again, since the median withstand the presence of errors, it will only affect if most of the samples have very low tumor purity which usually is not the case.

The number of samples used by NSA to build the reference is different for each locus. Therefore, the variance of the reference varies on each position. In particular, recurrently aberrated regions have a reference with larger variance than regions that show seldom aberrations.

Since NSA is based on finding regions enriched in HNCNs, if an aberration occurs in a region with no SNPs (it includes only CN probes), NSA cannot provide an accurate reference for this region.

Similarly, NSA estimates of chromosomes *X* and *Y * are not reliable for experiments that include only male samples. It is not useful either for experiments that include polyploid samples. These samples still pose a challenge for their analysis.

Finally, one potential situation in which NSA could fail is if *all* the samples present an aberration in the same locus. This is very unlikely event but it could potentially occur. Even in this putative case, a few samples hybridized in the same lab can be included in the study to avoid these “emergency cases”.

In spite of these limitations (most of them also present in other scaling methods), NSA is able to fix the problem of finding recurrent regions and copy number changes when no control samples are available.

If NSA is applied to a dataset with both tumoral and control samples, the detection of CN changes could be even more accurate than using MCS (especially if NSA removes the batch effects) because of having more and (what is more important) more appropriate samples to calculate the references. This fact makes NSA especially convenient to apply to many experiments (in GEO or ArrayExpress) without the need to explicitly state which are the control samples or the batches in the datasets.

In conclusion, NSA can be used to accurately scale summarized SNP signals to CNs. It presents less bias and noise than MTS or MHS and needs no control sample hybridization.

## Availability and requirements

The proposed NSA method is available in the NSA package implemented in R (R Development Core Team, 2010). This package includes an add on to the high-level aroma.affymetrix framework [33], which allows NSA to be applied to very large SNP data sets. It is publicly available at CRAN repository in a package called “NSA”.

## Abbreviations

LH, Level of Heterozygosity; HNCNs, Heterozygous Neutral Copy Number 775 SNPs; CNA, Copy Number Aberration; CNs, DNA Copy Numbers; SNP, Single 776 Nucleotide Polymorphism; LOH, Loss of Heterozygosity; CNVs, Copy Number 777 Variations; NSA, Normality Search Algorithm; BER, Batch Effect Removal; QP, 778 Quadratic Programming; MCS, Median of Control samples; MHS, Median of 779 HapMap samples; MTS, Median of tumoral samples.

## Competing interests

T he authors declare that they have no competing interests.

## Author’s contributions

MO conceived the idea and jointly with AA developed the add-on to the aroma.cn framework. AR developed the algorithm to account for batch effects. MO, AA and AR wrote the manuscript and developed the software to compare NSA against other algorithms. All authors read and approved the final manuscript.
